# Correlation between kinematic sagittal parameters of the cervical lordosis or head posture and disc degeneration in patients with posterior neck pain

**DOI:** 10.1515/med-2021-0219

**Published:** 2021-01-22

**Authors:** Hyo Jeong Lee, Dae Geun Jeon, Jung Hyun Park

**Affiliations:** Department of Rehabilitation Medicine, Gangnam Severance Hospital, Rehabilitation Institute of Neuromuscular Disease, Yonsei University College of Medicine, Seoul, Republic of Korea

**Keywords:** cervical vertebrae, intervertebral disc degeneration, kyphosis, lordosis, neck pain

## Abstract

The purpose of this retrospective cross-sectional study was to examine the degrees of the cervical disc degeneration and the parameters of cervical sagittal balance in plain radiographs, representing cervical lordosis or head posture in subjects with posterior neck pain. A total of 113 patients with posterior neck pain with or without radiating pain were analyzed. The kinematic sagittal parameters of cervical radiographs were obtained at the occipito–cervical (O–C2) angle, sagittal Cobb’s angles of C1–C2, C2–C7, and sagittal vertical axis (SVA) of C1–C7 and C2–C7. Cervical disc degeneration was evaluated using the sum of Pfirrmann grades and total modified Matsumoto scores calculated from MRI of the cervical spine. A significant positive correlation was found for the C2–C7 angle using the sum of the Pfirrmann grades and total modified Matsumoto scores, whereas the O–C2 angle and the C1–C2 angle were negatively correlated. The sagittal cervical parameters representing cervical kyphosis and jaw lifting posture were found to be significantly correlated with the degree of cervical disc degeneration. These findings suggest that the loss of the natural sagittal lordosis of the cervical spine may contribute to the progression of disc degeneration, rather than the forward head posture.

## 1 Introduction

Loss of cervical lordosis is the most common disorder of sagittal cervical balance [1,2]. Although the sagittal alignment of the cervical vertebrae can vary with age and sex [1,3], the natural sagittal curve of the cervical spine is known to have a lordosis [4,5,6]. Harrison et al. reported a mean C2–C7 lordotic angle of −26.89° ± 9.72° in 72 healthy participants [5]. Liu et al. demonstrated a mean C2–C7 lordotic angle of −21.40° ± 12.15° in 212 asymptomatic volunteers [6]. The estimated prevalence of loss of cervical lordosis in patients with posterior neck pain is approximately 42% [7]. Recently, several studies have demonstrated that a spectrum of cervical disorders are associated with a loss of cervical lordosis, i.e., kyphosis [1,8,9,10]. A cross-sectional study revealed that decreasing natural cervical lordosis was correlated with increasing Neck Disability Index scores in preoperative subjects [11]. Another prospective cohort study reported that patients with higher preoperative lordotic angles showed better outcomes than those with a kyphotic alignment [10]. Some studies have shown negative consequences of cervical malalignment on the health-related quality of life [1,12,13].

Forward head posture (FHP) is common in sagittal cervical imbalance in patients with symptomatic neck pain. FHP is usually defined as an increased sagittal vertical axis (SVA) in which the head is shifted anteriorly to the shoulder plane compared to the neutral posture. The normal physiological C2–C7 SVA is estimated to be 16.8 ± 11.2 mm in asymptomatic subjects [14]. Several studies have found that an increased SVA value is related to clinical symptoms [15,16].

The majority of research on sagittal cervical balance has been focused on analyzing radiographs of the cervical spine and comparing them with clinical symptoms [1,15,17], whereas few studies have focused on the relationship between cervical sagittal alignment and disc degeneration as seen on magnetic resonance imaging (MRI). Previously, the progression of cervical disc degeneration was higher in the non-lordotic group than in the lordotic group; however, the study only analyzed the C2–C7 Cobb’s angle regardless consideration of the other sagittal variables, such as SVA or the occipito–cervical angle [18]. Furthermore, few studies have analyzed the relationship between cervical degeneration, lordosis, and FHP at once.

The results of this study may serve as an investigation of the relationship between cervical disc degeneration as assessed using MRI and parameters of sagittal cervical balance in plain radiographs, representing cervical lordosis or FHP in patients with posterior neck pain. Therefore, it is possible to suggest selective muscle strengthening and rehabilitation exercise.

## 2 Methods

### 2.1 Subjects

A total of 421 patients with posterior neck pain over 3 months who visited the spine center of a university hospital in a metropolitan area were retrospectively analyzed. Only subjects with both cervical spine radiographs and cervical MRIs were included. The exclusion criteria were as follows: history of cervical spinal surgery or trauma, age under 19 years, symptoms or signs of inflammatory back pain, such as ankylosing spondylitis, imaging evidence of concurrent myelopathy, and spine fracture. A total of 113 patients were included in our study population. Informed consent was obtained from all individuals included in this study.

### 2.2 Measurements

#### 2.2.1 Kinematic parameters of cervical spine using plain radiographs

**Figure 1:**
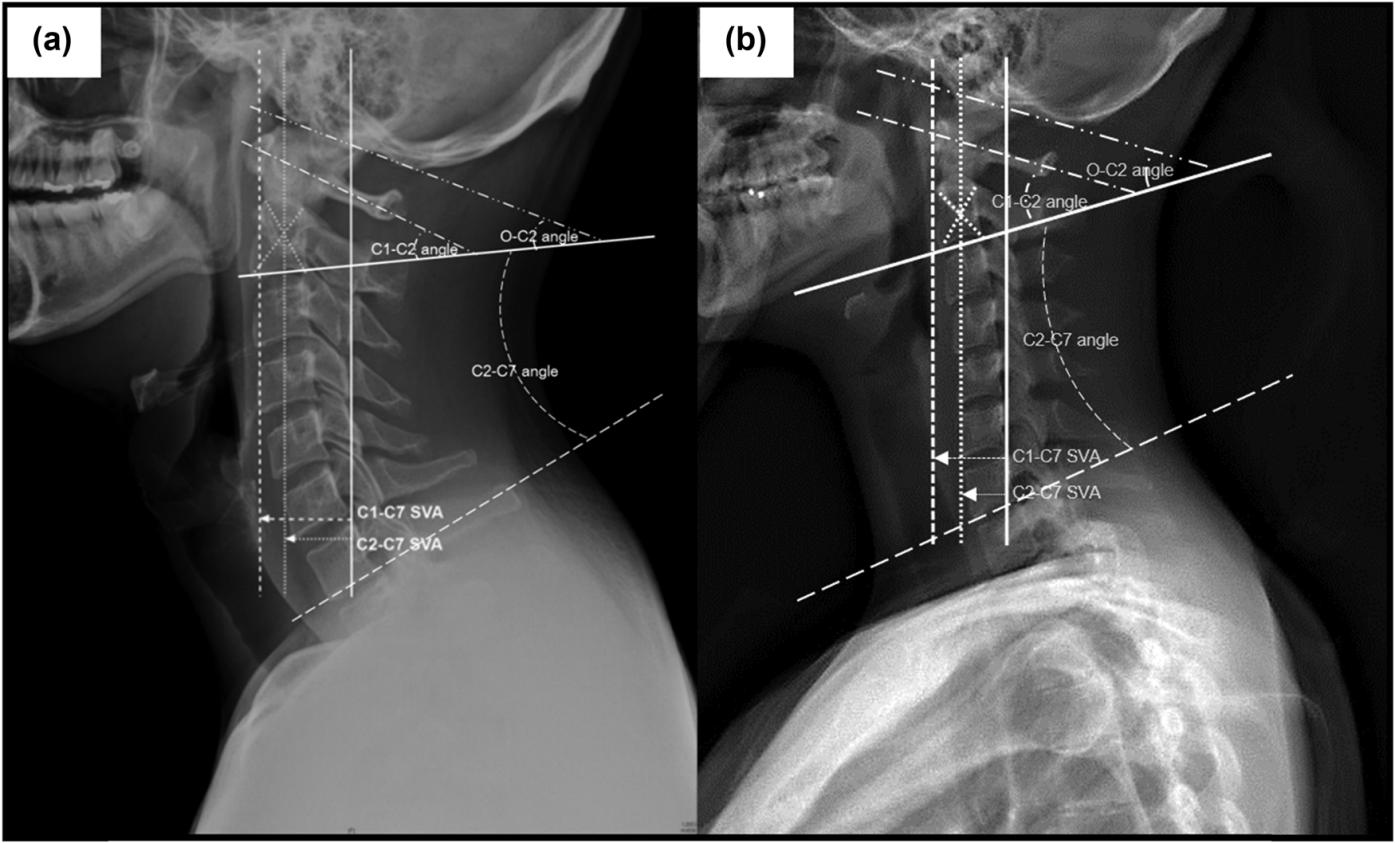
Parameters of sagittal cervical alignment on plain radiographs. (a) The lordotic group: O–C2 angle, angle between the McGregor line and the lower endplate of C2; C1–C2 angle, angle between a line connecting the anterior tubercle to the posterior margin of the C1 spinous process and the lower endplate of C2; C2–C7 angle, angle between the lower endplate of C2 and C7 determined with Cobb’s method; C1–C7 SVA, distance between the plumb line from the anterior arch of C1 and the posterior-superior corner of C7; C2–C7 SVA, distance between the plumb line from the centroid of C2 and the posterior-superior corner of C7. (b) The non-lordotic group.

All cervical plain radiographs were reviewed and analyzed using a picture archiving and communication system. Using plain lateral cervical radiographs, the following parameters were measured: occipito–cervical angulation (O–C2 angle), sagittal Cobb’s angles of the C1–C2, C2–C7 (C1–C2 angle, C2–C7 angle), and SVA of C1–C7, C2–C7 (C1–C7 SVA, C2–C7 SVA; Figure 1).

#### 2.2.2 Cervical disc degeneration measured by MRIs

3.0-T cervical MRIs (Discovery MR750; GE Healthcare, Milwaukee, WI) were performed to evaluate disc degeneration. Sagittal and axial T2-weighted MR imaging was performed for each cervical level. The severity of disc degeneration was assessed according to the Pfirrmann grades [19] and modified Matsumoto classification scores [20]. Each grade and score of the cervical levels from C2–C3 to C6–C7 was measured, and summation analysis was performed. Pfirrmann grades are used to evaluate degenerated intervertebral discs for (i) distinction of the annulus and the nucleus, (ii) disc structure, (iii) signal intensity of discs, and (iv) height of discs using T2-weighted mid-sagittal images [19]. The modified Matsumoto classification scores assess disc degeneration for (i) change in the signal of the disc, (ii) posterior protrusion of the disc, and (iii) narrowing of disc space in T2-weighted axial and mid-sagittal images [18].

#### 2.2.3 Lordotic and non-lordotic groups

To identify the specific correlation of kinematic sagittal parameters with cervical disc degeneration, the patients were divided into two groups (lordotic group and non-lordotic group; Figure 1), according to the C2–C7 Cobb angle observed on cervical plain radiographs. We set the cutoff of the C2–C7 lordotic angle as −17.17°, based on the study by Harrison et al. [5].

### 2.3 Statistical analysis

The comparisons between groups were performed using an independent *t*-test. The reliability of the measurements was assessed by examining the intra-observer and inter-observer reliability with intra-class correlation coefficients. The correlations between cervical parameters were determined using a Pearson’s test. A linear regression method was used to determine associations between the parameters of sagittal cervical alignment and the degree of cervical disc degeneration. All statistical analyses were performed with SAS version 9.3 (SAS Institute, Cary, NC).

### 2.4 Ethics statement

The research related to human use has been complied with all the relevant national regulations, institutional policies, and in accordance the tenets of the *Helsinki Declaration*, and has been approved by authors’ institutional review board.

## 3 Results

### 3.1 Basic characteristics of the two groups

This study included a total of 113 patients (55 males, 58 females). The baseline characteristics of the participants are summarized in Table 1. Twenty-six patients (10 males, 16 females) were classified into the lordotic group, and 87 patients (45 males, 42 females) were classified into the non-lordotic group. There were no statistical differences in age and sex between the two groups.

**Table 1:** Baseline demographic characteristics of participants

	Total participants (*n* = 113)	Lordotic group (*n* = 26)	Non-lordotic group (*n* = 87)
Age (years)	51.3 ± 10.1	52.4 ± 11.1	51.0 ± 9.8
**Sex**			
Male/female, *n* (%)	55 (48.6)/58 (51.3)	10 (38.4)/16 (61.6)	45 (51.7)/42 (48.3)
**Sagittal parameters**			
O–C2 angle (°)	−23.36 ± 7.40	−18.08 ± 5.85*	−24.94 ± 7.10
C1–C2 angle (°)	−26.27 ± 6.53	−22.4 ± 6.58*	−27.42 ± 6.09
C2–C7 angle (°)	−9.97 ± 10.00	−21.48 ± 5.78*	−6.53 ± 8.29
SVA of C1–C7 (mm)	26.83 ± 11.65	22.61 ± 8.76*	28.09 ± 12.15
SVA of C2–C7 (mm)	18.23 ± 9.43	16.03 ± 7.59	18.88 ± 9.85
**Cervical disc degeneration**			
Sum of Pfirrmann grades	14.7 ± 4.7	11.0 ± 4.6*	15.9 ± 4.2
Total modified Matsumoto scores	13.1 ± 4.3	9.92 ± 3.7*	14.0 ± 4.0

Values are presented as mean ± standard deviation.

SVA, sagittal vertical axis.

*P < 0.05, significant difference compared with the non-lordotic group.

### 3.2 Reliability analysis

The intra-rater and inter-rater observer agreements for cervical parameters and disc degeneration were analyzed (Table 2). In both intra-rater and inter-rater studies, good agreements were found, with *κ* values ranging from 0.72 to 0.91.

**Table 2:** Intra-rater and inter-rater reliability for all subjects

	Intra-rater reliability	Inter-rater reliability
O–C2 angle	0.89	0.81
C1–C2 angle	0.72	0.78
C2–C7 angle	0.88	0.85
C1–C7 SVA	0.89	0.87
C2–C7 SVA	0.85	0.91
Sum of Pfirrmann grades	0.88	0.85
Total modified Matsumoto scores	0.90	0.86

SVA, sagittal vertical axis.

### 3.3 Analysis of cervical angles

The C2–C7 angle was significantly correlated with the O–C2 and C1–C2 angles. The O–C2 angle was significantly correlated with the C1–C2 angle, C1–C7 SVA and C2–C7 SVA. The C1–C7 SVA and C2–C7 SVA were correlated with the C1–C2 angle (Table 3). There was no significant correlation between age and any measured radiographic parameter.

**Table 3:** Correlation among the cervical sagittal parameters

	C2–C7 angle	O–C2 angle	C1–C2 angle	C1–C7 SVA
O–C2 angle	−0.445^**^			
C1–C2 angle	−0.322^**^	0.724^**^		
C1–C7 SVA	0.294	−0.560^**^	−0.339^**^	
C2–C7 SVA	0.198	−0.505^**^	−0.302^**^	0.974^**^

*Correlation was significant at the *P* < 0.05 level. **P < 0.01 level.

SVA, sagittal vertical axis.

### 3.4 Analysis of cervical disc degeneration by Pfirrmann grades

**Figure 2:**
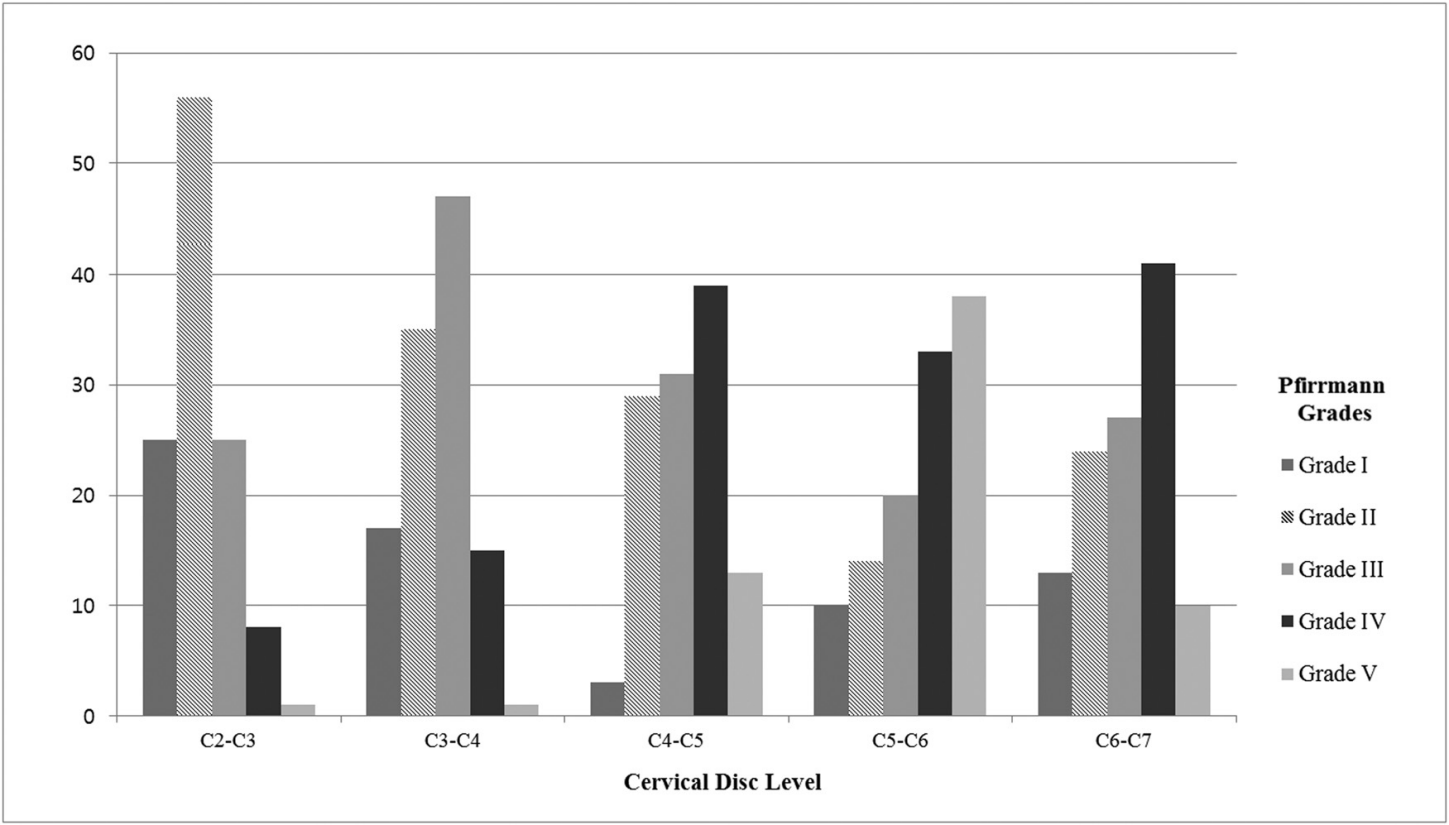
Number of patients with cervical disc degeneration assessed using Pfirrmann grades.

The degree of disc degeneration was assessed using Pfirrmann grades at each level. In the assessment of disc degeneration according to Pfirrmann grades, grade I was most commonly observed at the C2–C3 level. Grade V was more common at the C5–C6 level than at any other level (Figure 2).

### 3.5 Correlation between kinematic parameters of the cervical spine and disc degeneration

#### 3.5.1 Cervical angles and disc degeneration

**Figure 3:**
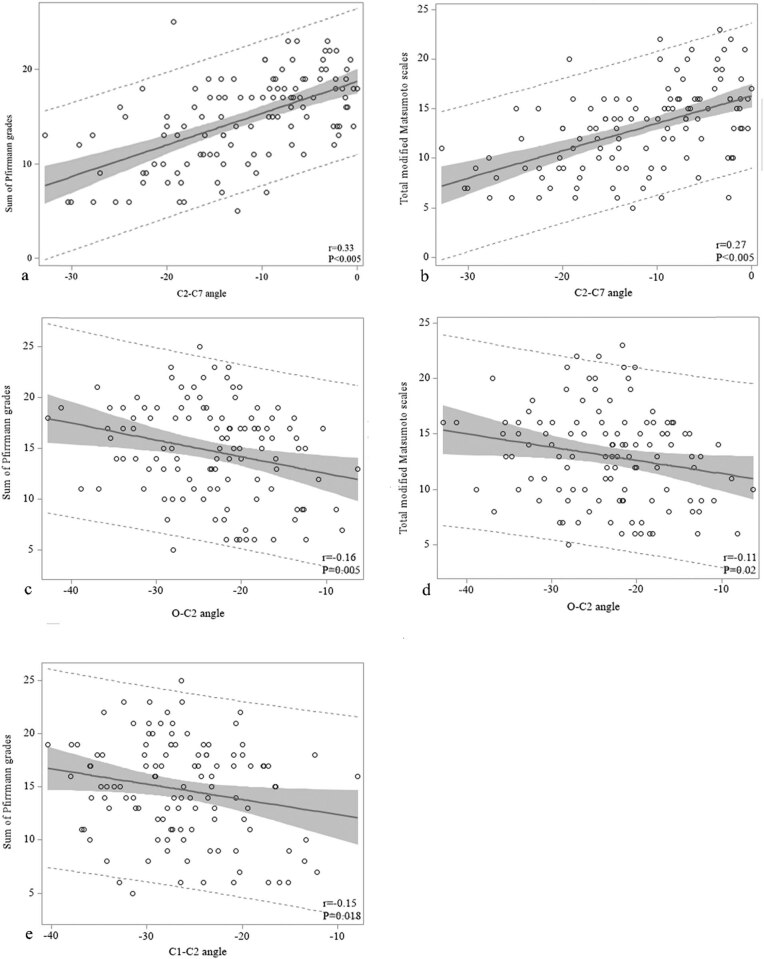
Correlation between sagittal angles of the cervical spine and the degree of cervical disc degeneration. (a) The C2–C7 angle and sum of Pfirrmann grades; (b) the C2–C7 angle and total modified Matsumoto scales; (c) the O–C2 angle and sum of Pfirrmann grades; (d) the O–C2 angle and total modified Matsumoto scales; and (e) the C1–C2 angle and sum of Pfirrmann grades.

Using a multivariate regression analysis adjusted for age, the C2–C7 angle revealed a significant positive correlation with both the sum of Pfirrmann grades (*r* = 0.33, *P* < 0.005) and the total modified Matsumoto scores (*r* = 0.27, *P* < 0.005), although age was not a co-linear factor (Figure 3a and b). There was a significant negative correlation between the O–C2 angle, and both the sum of Pfirrmann grades (*r* = −0.16, *P* = 0.005) and the total modified Matsumoto scores (*r* = −0.11, *P* = 0.02) (Figure 3c and d). The C1–C2 angle was negatively correlated with the Pfirrmann grades (*r* = −0.15, *P* = 0.018) (Figure 3e).

#### 3.5.2 SVA and disc degeneration

The C2–C7 SVA and the C1–C7 SVA did not reveal a correlation with the sum of Pfirrmann grades or the total modified Matsumoto scores (Table 4). In both subgroups (lordotic and non-lordotic groups), the SVA had no significant correlation with the Pfirrmann grades or the total modified Matsumoto scores (Table 4).

**Table 4:** Correlation between sagittal cervical vertical axis and degree of cervical disc degeneration

		Total participants (*n* = 113)		Lordotic group (*n* = 26)		Non-lordotic group (*n* = 87)	
		C1–C7 SVA	C2–C7 SVA	C1–C7 SVA	C2–C7 SVA	C1–C7 SVA	C2–C7 SVA
Sum of Pfirrmann grades	Coefficient	0.064	0.063	−0.027	−0.006	0.038	0.042
	*P*	0.088	0.18	0.837	0.960	0.301	0.347
Total modified Matsumoto scores	Coefficient	0.049	0.044	0.017	0.013	0.020	0.022
	*P*	0.139	0.281	0.821	0.881	0.565	0.609

SVA, sagittal vertical axis.

## 4 Discussion

The cervical spine naturally maintains a lordotic curvature to compensate for the thoracic kyphotic curvature [3]. As the loss of cervical lordosis progresses, the deformity also tends to progress rapidly by producing abnormal forces to the head and neck [1,13]. Even with mild sagittal imbalance, detrimental symptoms can develop, which worsen with sagittal imbalance progression [21]. The vertebral disc is designed to maintain an isotropic form by transmitting axial load uniformly across the disc and vertebral endplate [22,23]. In cervical spinal positions, such as extension, flexion, or lateral bending, the load of the disc is transmitted uniformly over the endplates [23]. Loss of cervical lordosis may alter this isotropic nature of disc loading and consequently contribute to continuous irregular loading, which accelerates disc degeneration [23,24]. This degeneration can be aggravated by normal aging, calcification of the endplate, or decreased peripheral blood supply [25]. Abnormally increased mechanical pressure has also been shown to reduce the nutritional support of the disc and lead to disc degeneration [26]. Our results demonstrate a correlation between loss of cervical lordosis and cervical disc degeneration. In this study, cervical disc degeneration was evaluated using both Pfirrmann grades and the modified Matsumoto scoring system, whereas most previous studies have only used either of them [18,20,27,28]. Pfirrmann grading assesses the homogeneity of disc structure and includes a distinction between the annulus and nucleus, whereas the modified Matsumoto scoring system considers the degree of the posterior disc protrusion and narrowing of disc space without considering the homogeneity of the disc. This difference in the evaluation criteria may explain the different relationship between the C1–C2 angle and disc degeneration, so the C1–C2 angle is only analyzed to the Pfirrmann grades.

Changes in sagittal cervical alignment, such as FHP, may cause or result in adaptive mechanisms to global alignment change, which affects all spinal levels (including the cervical, thoracic, and lumbar regions) [1,29]. Contractions of the neck muscles because of vestibulocollic or cervicocolic reflexes induce anterior shifting of the head/neck center of gravity, resulting in a change in the spinal alignment [30,31]. These reflexes cause cervical muscle spasm, representing shortening of the posterior neck extensor muscles and the tightening of the anterior neck muscles, which may increase the SVA [32]. Previous studies have shown that a larger C2–C7 SVA is related to higher Neck Disability Index scores and demonstrated a correlation between the C2–C7 SVA and the C1–C2 angle [16]. The results of this study also showed that the C2–C7 SVA was significantly correlated with the O–C2 angle and the C1–C2 angle. However, to date, the clinical consequences of increased cervical SVA on cervical disc degeneration have not been described. In this study, the change in SVA was not correlated with cervical disc degeneration. In both the lordotic and non-lordotic groups, the SVA was not correlated with cervical disc degeneration. These findings indicate that the SVA, which has been suggested to be related to FHP in previous studies, has little effect on disc degeneration. Cervical disc degeneration progressing with normal aging could be aggravated by the loss of natural sagittal angles rather than increased cervical SVA. Recently, one study showed significant correlation between cervical lordosis and C2–C7 SVA in asymptomatic Chinese population [33]. In this study, there was no significant correlation between C2–C7 angle and C2–C7 SVA. This may mean that the rate of disc degeneration is greater than that of FHP when clinical symptoms occur.

The optimal position and angle of the occipital bone and the cervical axis have been topics of discussion [34,35,36]. In this study, there was a negative correlation between the O–C2 and C1–C2 angles and the C2–C7 angle, as well as a significant correlation with the SVA. These results suggest a correlation between the occipital–cervical axis, cervical kyphosis, and FHP. There was a significant correlation between the occipito–cervical angle and cervical disc degeneration in this study. A recent cohort study showed that an increased occipito–cervical angle may result in large biomechanical stress on the adjacent structures or deformation of cervical alignment [35]. A previous study has shown that the loss of the natural C2–C7 angle facilitates cervical disc degeneration [18]. In addition, our findings suggest that a more negative occipito–cervical angle may accelerate disc degeneration. In summary, the SVA, the occipito–cervical angle, and the loss of cervical lordosis expressed easily as the “FHP,” “the jaw lifting posture,” and “the cervical kyphosis” were correlated with each other. In addition, only the latter two, the jaw lifting posture and the cervical kyphosis, were correlated with disc degeneration. Although the cause-and-effect relationship is unknown, it can be interpreted that FHP worsens the jaw lifting posture and cervical kyphosis, which may cause disc degeneration.

This study has some limitations. The pathophysiological mechanism of disc degeneration because of the loss of cervical lordosis remains unknown. As the analyses were cross-sectional, determining a cause-and-effect relationship is difficult. Furthermore, neck pain was not classified as causal factor in this study. As cervical disc degeneration progresses, neck pain or uneven loading to the cervical disc can induce deformity of the sagittal alignments, including both cervical and occipito–cervical angles. Long-term changes in the sagittal cervical parameters and disc degeneration were also not evaluated. Prospective longitudinal studies with long-term follow-up and larger sample sizes are necessary to investigate the clinical implications and the interactions between the alignment of cervical spines and the discs. In addition, further study will be necessary to subdivide the basic characteristics, such as body weights, body mass index, and work style. Finally, as we did not include an asymptomatic group of participants, the results may not be generalizable to whole populations. Future long-term longitudinal studies in a general asymptomatic population are needed.

In conclusion, this study showed that sagittal cervical parameters, such as the C2–C7 angle, O–C2 angle, and C1–C2 angle, were correlated with the degree of cervical disc degeneration in patients with posterior neck pain. However, there was no significant correlation between the SVA and disc degeneration. These results revealed that loss of the natural cervical lordosis is correlated substantially with cervical disc degeneration, rather than the FHP.
